# Evaluating the burden of endogenous Cushing’s syndrome using a web-based questionnaire and validated patient-reported outcome measures

**DOI:** 10.1007/s11102-023-01314-7

**Published:** 2023-04-19

**Authors:** Gabrielle Page-Wilson, Bhagyashree Oak, Abigail Silber, Janetricks Okeyo, Nancy Ortiz, Matthew O’Hara, Stephen Moloney, Eliza B. Geer

**Affiliations:** 1 Division of Endocrinology, Columbia University, New York, USA; 2 Trinity Life Sciences, Waltham, USA; 3 Formerly at Strongbridge Biopharma plc, a wholly owned subsidiary of Xeris Biopharma Holdings, Inc, Trevose, USA; 4 Multidisciplinary Pituitary and Skull Base Tumor Center, Memorial Sloan Kettering Cancer Center, 1275 York Avenue, New York, NY 10065 USA

**Keywords:** Cushing’s syndrome, Cushing’s disease, Health-related quality of life, Patient reported outcomes, Healthcare resource utilization, Burden of illness

## Abstract

**Introduction:**

Endogenous Cushing’s syndrome (CS) is a rare endocrine condition caused by chronic oversecretion of cortisol, resulting in a diverse constellation of symptoms. This study examined the ongoing burden of illness (BOI), from the first appearance of symptoms through treatment, which is currently not well evaluated.

**Methods:**

A quantitative, cross-sectional, web-enabled survey including 5 validated patient reported outcomes (PRO) measures was conducted in patients with CS who had been diagnosed ≥ 6 months prior and who had received ≥ 1 treatment for their endogenous CS at the time of the survey.

**Results:**

Fifty-five patients participated in this study; 85% were women. The mean age was 43.4 ± 12.3 years (± standard deviation, SD). On average, respondents reported a 10-year gap between the first occurrence of symptoms and diagnosis; 80% underwent surgical treatment for CS. Respondents experienced symptoms on 16 days in a typical month, and their health-related quality of life was moderately impacted based on the CushingQoL score. Weight gain, muscle fatigue, and weakness were the most common symptoms and 69% percent of patients reported moderate or severe fatigue using the Brief Fatigue Inventory. Following treatment, the occurrence of most symptoms declined over time, although anxiety and pain did not significantly decrease. Overall, 38% of participants reported an annual average of 25 missed workdays due to CS symptoms.

**Conclusions:**

These results demonstrate a BOI in CS despite ongoing treatment and illustrate the need for interventions to address persistent symptoms, particularly weight gain, pain, and anxiety.

## Introduction

Endogenous Cushing’s syndrome (CS) is a rare, debilitating disorder caused by chronic oversecretion of cortisol, that results from adrenocorticotropic hormone (ACTH)-secreting pituitary or ectopic tumors or from cortisol producing adrenal tumors. [1] Multiple comorbidities are associated with CS including cardiovascular disease, metabolic disorders, musculoskeletal, and mental health conditions. The associated symptoms and comorbidities lead to a health-related quality of life (HRQoL) that is notably worse than population norms. [2].

Reducing the morbidity and mortality associated with CS requires both early diagnosis and treatment, and attention to the long-term clinical sequalae. Unfortunately, the clinical diagnosis may be challenging because CS is characterized by a constellation of symptoms that often overlap with common conditions such as obesity, metabolic syndrome, polycystic ovary syndrome, and depression. As a result, the time from initial appearance of symptoms to diagnosis is, on average, 3 years. [3] Furthermore, potentially irreversible comorbidities including cardiovascular and neurocognitive disorders, have been associated with long-term exposure to elevated cortisol due to untreated CS. [3, 4] Even when the underlying cause of hypercortisolism has been diagnosed and treated, CS has been associated with long-term reductions in HRQoL. [5, 6].

First-line treatment of CS is surgical resection of the causative tumor. Radiotherapy and medical therapy may be used second line in patients for whom surgery is unsuccessful, who are not surgical candidates, or who do not achieve surgical remission. [4] Tumor-directed therapies including the somatostatin analog pasireotide and the dopamine agonist cabergoline, steroidogenesis inhibitors, and the glucocorticoid receptor antagonist mifepristone are also available for use pre-operatively, when awaiting the effects of radiotherapy, or in the case of disease recurrence. [2, 4, 7] In patients with pituitary Cushing’s disease (CD), which represents 70% of all cases, medical therapy has been reported to result in adequate control of cortisol levels in 32–84% of patients; severe side effects can cause up to 1/3 of patients to adjust or discontinue medical treatment. [8, 9] Although achieving biochemical control can improve some of the symptoms of CS by mitigating the underlying hypercortisolism, residual symptoms and medication related side effects may continue to erode HRQoL. [8, 10].

Previous studies exploring the burden of illness (BOI) of CS have focused solely on patients with either ACTH dependent CS or ACTH independent CS, and not on the collective burden of all CS patients, limiting the generalizability of the results. [11–13] Currently, there is a dearth of research examining the patient reported BOI using multiple patient reported outcomes (PRO) measures. The overall objective of this study was to evaluate the long-term burden of CS before and after treatment using a comprehensive questionnaire as well as disease and symptom specific validated PRO measures to analyze treatment related changes in the disease burden and the performance of current therapies. In addition to characterizing the symptomatic burden of CS, this study aimed to describe the impact of CS on activities of daily living, including healthcare resource utilization and work productivity. This study may not only identify targetable symptoms that can inform the future treatment landscape for CS but may also guide future research on the potential use of PRO measures in relevant clinical practice and research settings.

## Methods

### Study design and recruitment

This quantitative, cross-sectional survey was conducted to collect BOI data from qualified patient respondents. The study protocol was granted exempt status by ADVARRA IRB (Columbia, MD; https://www.advarra.com/). The study instrument included a patient screener and a web-enabled questionnaire designed to collect information on the BOI across multiple dimensions including clinical, economic, psychological, social, and HRQoL domains via quantitative and numerically based responses.

Patients were recruited via patient panels and physician-assisted recruitment via third-party vendors and by utilizing an IRB-approved study flyer with relevant patient advocacy groups. The study was fielded from June 1 through August 31, 2021.

This was an observational study and not a randomized clinical trial, and therefore was not registered as such.

### Patient eligibility criteria

The eligibility of each respondent was confirmed via screening questions that preceded the main questionnaire. Eligible patients were at least 21 years old and reported having a physician-confirmed diagnosis of CS. In addition, patients received their diagnosis at least six months before the study, had received at least one pharmacologic therapy to treat CS, and were receiving pharmacologic therapy as a treatment for their CS at the time of the study. Finally, eligible patients needed to be able to access the web-based survey via an internet-connected device and complete the survey in English. Potential respondents were excluded if they received a diagnosis of exogenous CS, had existing adrenal or pituitary carcinomas, or had participated in a clinical trial in the previous six months.

### Web-based questionnaire

The questionnaire was approximately 30 min in length, and was accessed via the respondents’ computer, tablet, or smartphone. Qualified respondents proceeded immediately from the screening questions to the main questionnaire. The questionnaire evaluated respondents’ symptoms, CS experience, their treatment history, impact the condition had on their HRQoL using multiple validated PROs, healthcare resource utilization (HCRU), and work productivity The type and intensity of symptoms due to CS were assessed in the survey at three different time points including: (1) at the first onset or recognition of symptoms, (2) at the time of initial diagnosis, and (3) at the time the survey was taken.

Five validated PROs were utilized in the survey including the CushingQoL, which is a validated, disease-specific instrument to assess HRQoL specifically in patients with CS. [6] To assess pain associated with CS, the questionnaire included a visual analog scale (VAS), a widely used unidimensional measure of pain intensity with a numerical range from 0 to 10, with higher scores indicating higher reported pain. [14] The Brief Fatigue Inventory (BFI), a 10-point scale ranging from 1 to 3 (mild), 4–7 (moderate), and 8–10 (severe), was used as a measure of the severity of fatigue. [15] Finally, two patient-centered measures from the Patient-Reported Outcomes Measurement Information System (PROMIS) were used. The Sleep Disturbance v1.0 scale was used to assess self-reported perceptions of sleep, while the PROMIS Short Form Anxiety v1.0–8a scale provided an 8-question evaluation of self-reported fear, anxious misery, hyperarousal, and somatic symptoms related to arousal. [16–18] These assessments are relevant across all conditions and can be used in both the general population and patients with chronic conditions. The mean score of the US general population is set to 50 for these assessments, which allows comparisons with other disease-specific populations; a score greater than 50 indicates greater sleep disturbance (Sleep Disturbance v1.0) or greater anxiety (PROMIS Short Form Anxiety v1.0).

Respondents were also asked to score, on a 7-point scale (with 1 indicating “no impact at all” and 7 indicating “very significant impact”), the impact of CS on activities of daily living. Likewise, the questionnaire queried respondents on their physician’s level of awareness about the impact of CS on a scale from 1 (not aware at all) to 7 (very aware).

### Statistical analysis

Descriptive statistics including means, standard deviations (SD), medians, and frequencies were used to describe the study population. Paired T-tests were conducted to analyze statistical significance of mean differences, as appropriate. Subgroup analyses were also performed with 95% confidence intervals calculated to allow for statistical comparison between subgroups. All statistical analyses were conducted using R statistical software (www.r-project.org/) and Q Research Software 5.6 (Q Research Software, New York, NY).

## Results

### Respondent characteristics

A total of 55 patients were enrolled with a mean age of 43.4 ± 12.3 years; 47 (85%) were women (Table 1). Respondents reported they first started experiencing symptoms of CS at a mean age of 30 ± 12.6 years, but the mean age at diagnosis was 40 ± 12.3 years. Of the 46 respondents who reported their height and weight, over 95% were obese (body mass index [BMI] ≥ 30) or overweight (25-29.9), and most patients had comorbidities including high blood pressure and diabetes or pre-diabetes, both of which affected 25 (45%) patients. [19] About a third of respondents (20 patients; 36%) required assistance from a caregiver (home health nurse, family member, or friend who provided support as needed) to manage their condition. About half (27 patients; 49%) of respondents did not work and just over one third (19 patients; 35%) were engaged in full-time employment at the time of the survey.

**Table 1:** Patient Demographics and Clinical Characteristics

	N = 55 unless otherwise noted
Age (Mean ± SD)	43.4 ± 12.3 years
Female N (%)	47 (85)
Age at first occurrence of CS symptoms (Mean ± SD)	30.3 ± 12.6 years
Age at diagnosis (Mean ± SD)	40.2 ± 12.3 years
Employment Status N (%)	
- Full-time - Part-time - Never employed - Inactive^1^ - Currently seeking employment/underemployed - Prefer not to say	19 (35) 8 (15) 8 (15) 18 (33) 1 (2) 1 (2)
Ethnicity N (%)	
- White or Caucasian - Black or African American - Hispanic or Latino - Asian - American Indian or Alaska Native	34 (62%) 10 (18) 8 (15) 2 (4) 1 (2)
Insurance Status (%)	
- Employer-provided private or commercial insurance - Medicaid - Medicare - Self-purchased private or commercial insurance - Other government insurance (i.e., VA, DOD)	24 (44%) 19 (35) 11 (20) 2 (4) 2 (4)
BMI Categories N (%) - Obese (≥ 30) - Overweight (25-29.9) - Normal or healthy weight (18.5–24.9) - Underweight (< 18.5)	N = 46 42 (91) 2 (4) 2 (4) 0 (0)
Comorbidities N (%)	
- High blood pressure - Diabetes or pre-diabetes - High cholesterol - Osteoporosis - Infertility - Cardiovascular disease/stroke - Blood clots (deep vein thrombosis/pulmonary embolism - None of the above	27 (49) 25 (45) 14 (25) 6 (11) 3 (5) 1 (2) 0 (0) 15 (27)
Caregiver Assistance Received N (%)	20 (36)
Types of Tumors N (%) - Adrenal carcinoma - Adrenal adenoma - Adrenal hyperplasia - Pituitary carcinoma - Pituitary adenoma - Pituitary hyperplasia	N = 44 0 (0) 10 (23) 11 (25) 0 (0) 19 (43) 9 (20)
Surgery status	
- Yes - No	44 (80) 11 (20)
Medications Received by Mechanism of Action N (%)	
- Steroidogenesis inhibitor - Glucocorticoid-receptor antagonist - Tumor-directed therapy - Chemotherapy - Other	29 (53) 10 (18) 28 (51) 2 (4) 1 (2)
Patients who discontinued at least one therapy N (%)	15 27)

^1^“Inactive” employment status includes participants who are retired, homemakers, disabled, and full- and part-time students

Of the 55 respondents, 45 (81%) had pituitary or adrenal tumors and 11 (20%) had ectopic ACTH producing tumors. Those with adrenal tumors had either adrenal adenomas (10 patients; 23%) or hyperplasia (11 patients; 25%), while the patients with pituitary-dependent disease had pituitary adenomas (19 patients; 43%) and pituitary hyperplasia (9 patients; 20%) (Table 1). Respondents were able to select all the options that were applicable to them for these questions. A majority of patients (44 patients; 80%) underwent surgery to treat their CS, with the most common surgeries being adrenal gland (19 patients; 35%) and pituitary tumor (16 patients; 29%) removal. Steroidogenesis inhibitors were the most common medication type (29 patients; 53%). The most common specific pharmacotherapy received by the 55 respondents was long acting pasireotide (14 patients; 25%), while 10 (18%) patients received the short-acting release formulation. The discontinuation rate for all pharmacotherapies was 35%, and at least half of the patients taking ketoconazole and cabergoline discontinued therapy.

### Symptomatic burden

**Fig. 1:**
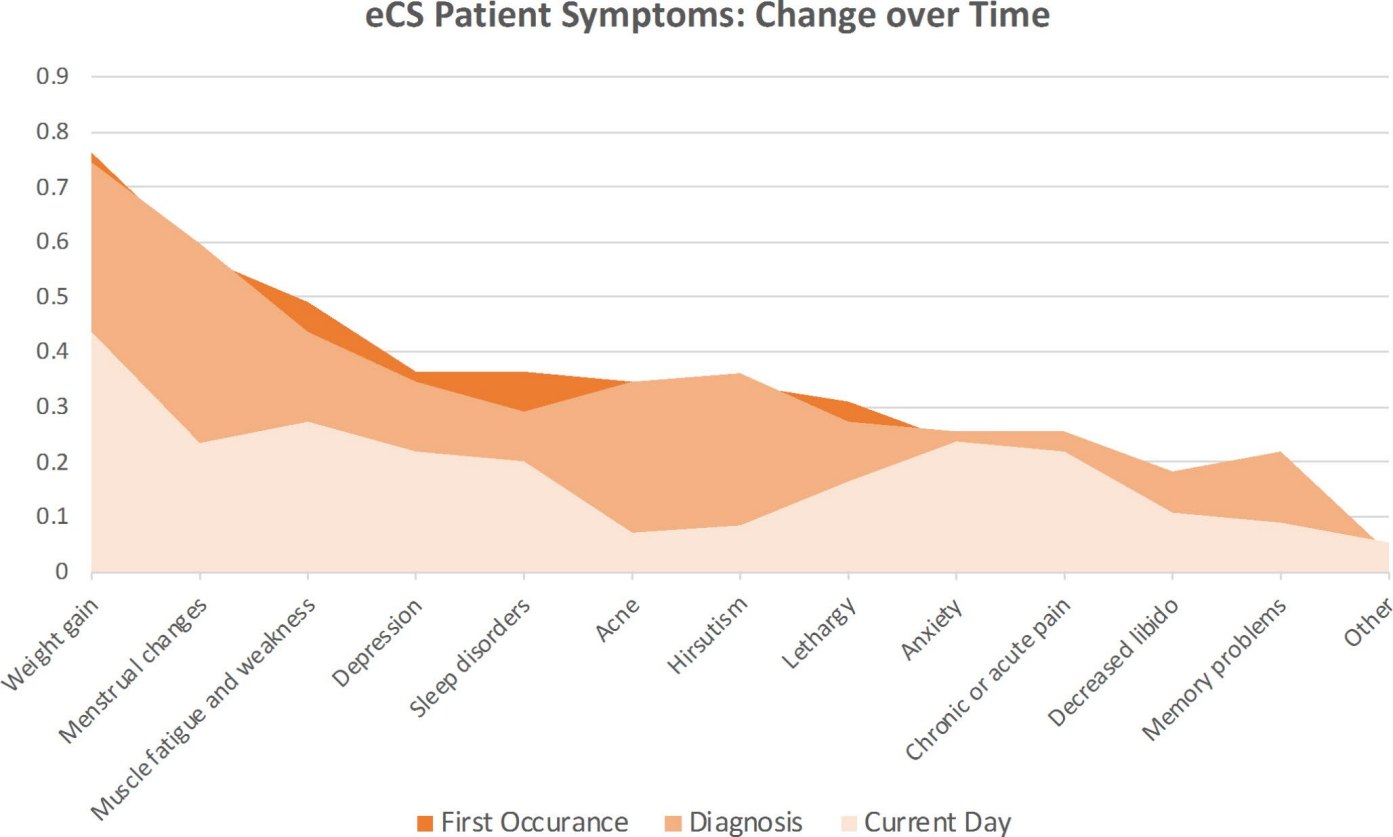
The frequency of reported symptoms at first occurrence, diagnosis, and the current time

Patients reported that the frequency of most symptoms declined from the first occurrence of symptoms to the time of the survey, although none of the symptoms were fully eliminated (Fig. 1).

There was no statistically significant difference in the frequency of reported symptoms over time. Notably, weight gain was reported by 76% of the 55 respondents at first occurrence of symptoms and by 75% at diagnosis, and this proportion declined to 44% by the time of the survey. Likewise, 49% of patients experienced muscle fatigue and weakness at first occurrence and 44% at diagnosis, but this percentage declined to 27% at the time of the survey. In contrast, anxiety was reported by the same proportion of respondents at first occurrence of symptoms and at the time of the survey (24%), and reported pain also remained essentially the same (24% at baseline to 22% at the present time).

**Fig. 2:**
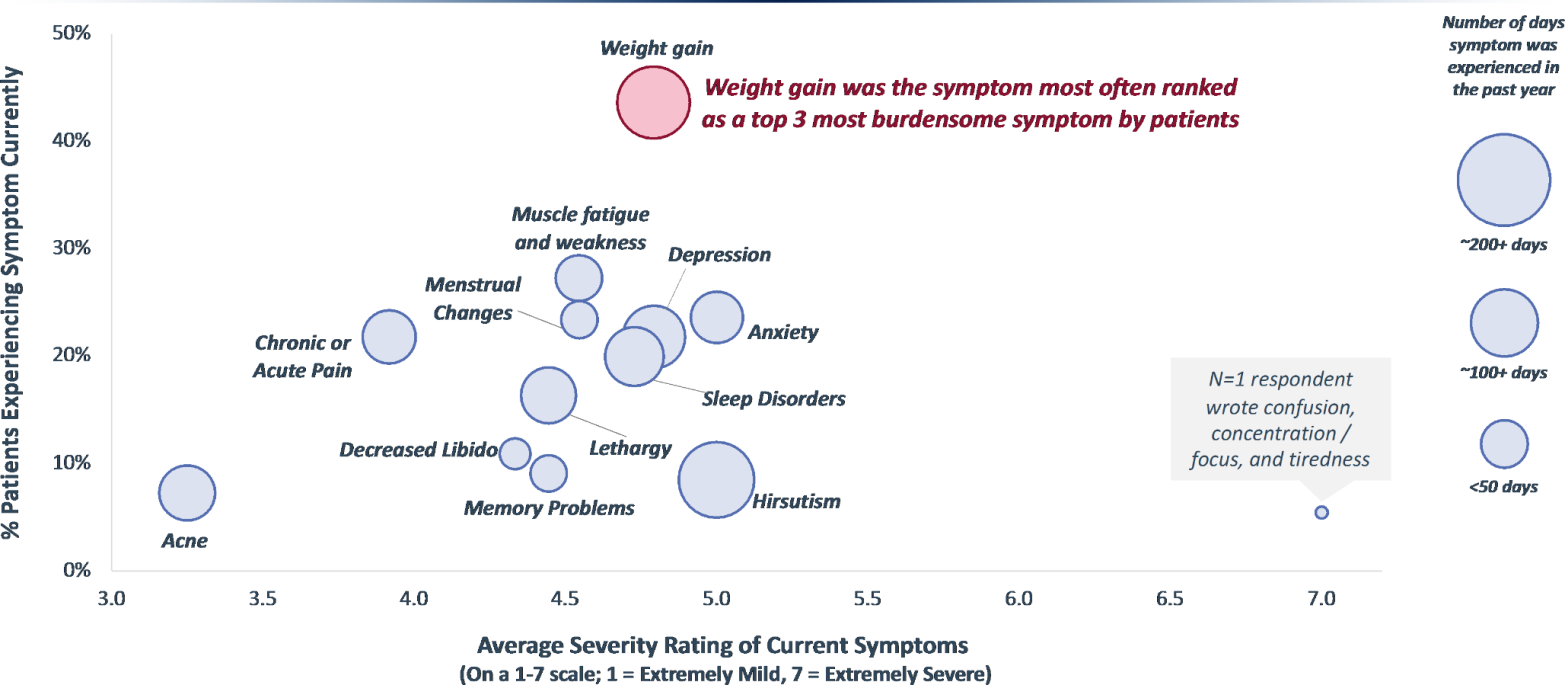
Current symptoms plotted by their severity (x-axis) and frequency (y-axis), and the size of the individual plot points is relative to the number of days the symptom was experienced in the past year

The symptomatic burden in this patient sample was substantial, with 41 of 55 respondents (75%) reporting 3–5 symptoms at the time of first occurrence, and 64% reporting 3–5 symptoms at the time of the survey. Only 4% of patients reported no symptoms at the current time. The 3 most frequently reported symptoms at the time of the survey included weight gain, muscle fatigue and weakness, and anxiety (Fig. 2).

When compared to symptom severity at diagnosis, declines in symptom severity at the time of the survey were observed for weight gain (5.8 ± 1.2 at diagnosis vs. 4.8 ± 1.3 at the time of the survey), muscle fatigue and weakness (5.7 ± 0.9 vs. 4.5 ± 1.5), and menstrual changes (5.5 ± 1.0 vs. 4.5 ± 1.4). Although these declines in symptom severity were statistically significant, these symptoms were not eliminated entirely. Declines in the severity of other symptoms, such as lethargy and depression were not statistically significant. Hirsutism and anxiety, while some of the most severe ongoing symptoms, were reported by very few patients at diagnosis (n = 17) and at the time of the survey (n = 4).On a scale from 1 to 7, hirsutism and anxiety consistently scored high at first occurrence, diagnosis, and at the time of the survey. Additionally, hirsutism was one of the few symptoms where symptom severity remained consistent at diagnosis (5.1) and after treatment at the time of the study (5.0), on average. Although some changes in symptom frequency and severity were observed, there was no statistically significant change in patient satisfaction with their medications from their first appointment to the current time.

### Diagnostic challenges

The mean age of initial onset of symptoms across study respondents was 30 ± 12.6 years, while the mean age at diagnosis was 40 ± 12.3 years (Table 1), indicating an average interval of a decade in which patients lived with undiagnosed CS. All 55 respondents in this study reported at least 1 challenge associated with receiving a confirmed diagnosis of CS (100%), and 24 respondents (44%) reported more than 1 challenge in receiving a diagnosis including 6 (11%) who reported 4 or more. The most common diagnostic challenge reported by 27 (49%) patients, was the inability of their primary care provider to diagnose respondents’ CS and 18 (33%) initially received the wrong diagnosis. About half (27 patients; 49%) of the 55 respondents reported being referred to a specialist by their original physician, and in addition, 39% of patients reported a lack of knowledge or understanding of the condition by their doctor as a key diagnostic challenge.

### Validated patient reported outcomes

Mean scores from validated patient reported outcomes (PRO) scales are reported in Table 2.

**Table 2:** Outcomes of Validated Patient-Reported Outcome (PRO) Measures

	Mean ± SD (N = 55)
CushingQoL Score	55.8 ± 20.4
Pain due to CS (VAS)	3.6 ± 1.9
Brief Fatigue Inventory	
- Mild - Moderate - Severe	31% 53% 16%
PROMIS Sleep Score	55.0 ± 5.4
PROMIS Anxiety Short Form (raw score)	57.6 ± 7.5

On average, the 55 respondents experienced moderate levels of impairment to their HRQoL due to CS, as assessed by a score of 55.8 ± 20.4 on the CushingQoL scale. The mean pain score (as measured by the VAS) of the cohort was reported as 3.6 out of 10, indicating relatively low levels of pain, although notably 89% of respondents reported taking over the counter (OTC) analgesics. 69% of the cohort reported moderate to severe levels of fatigue according to BFI scores. Mean scores on the PROMIS Sleep Disturbance and PROMIS Short Form Anxiety 8a assessments are also reported. A T-score of 50 is set to the mean response of the United States (US) general population. The mean scores of our respondents were within 1 standard deviation of 50 for both sleep and anxiety, and therefore were not significantly different from the general population. However, data on use of sleep aids, anti-anxiety medication, and sedative-hypnotics were not collected. [17] Overall, the PRO scales illustrated that patients were experiencing moderate to severe fatigue, moderate HRQoL interference, and mild pain.

### Impact on activities of daily living

**Fig. 3:**
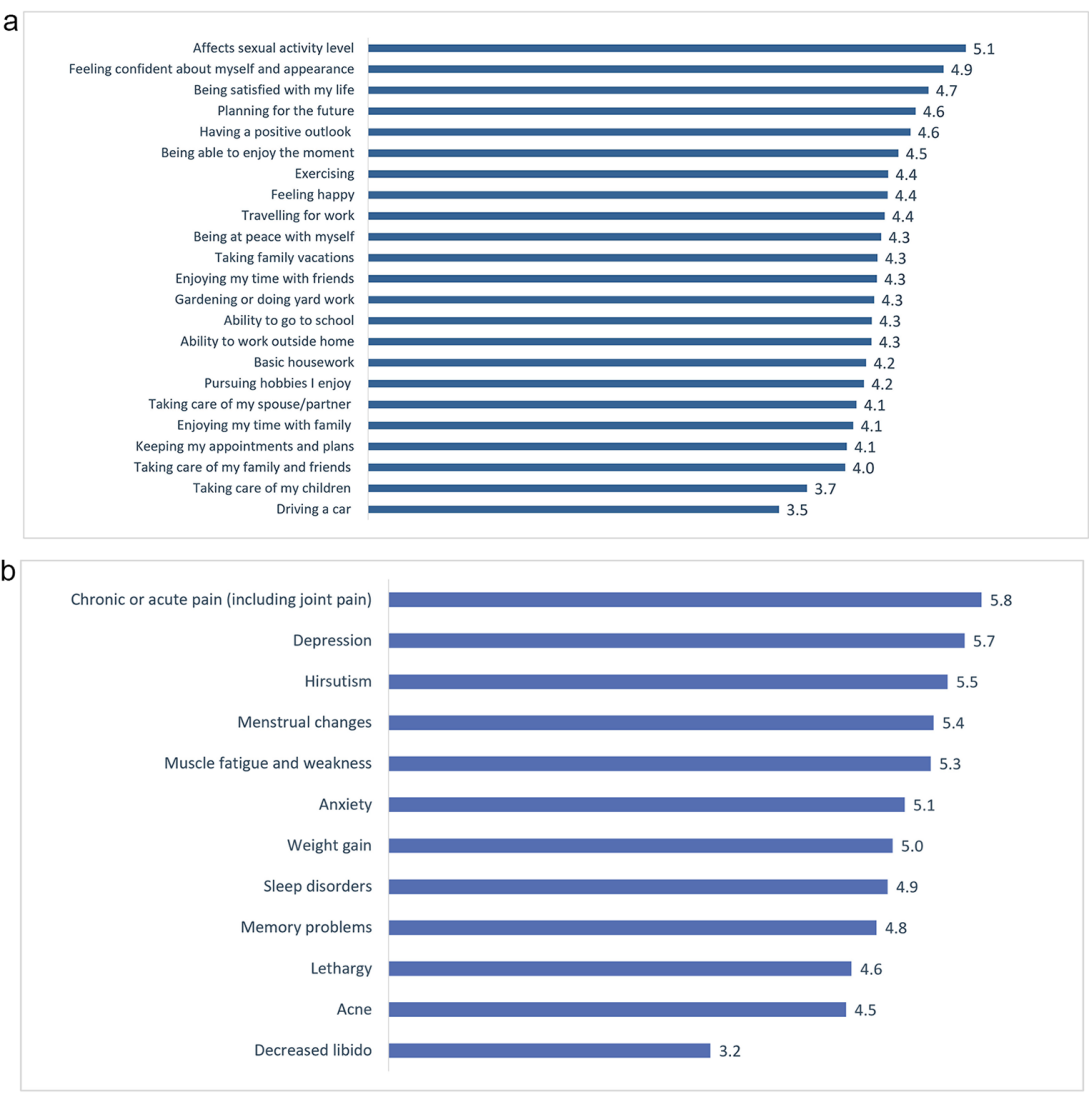
Panel **a** shows the impact of CS on activities of daily living reported by all respondents (N = 55) on a scale of 1–7, where 1 is “no impact at all” and 7 is a “very significant impact.” Panel **b**, physicians’ level of awareness of the impact of CS on patients’ daily activity is shown, as reported by all respondents (N = 55) on a scale of 1–7, where 1 is “not aware at all” and 7 is a “very aware”

The activities of daily living most impacted by CS were sexual activity, self-confidence, and life satisfaction (Fig. 3). Notably, respondents reported that their physicians were most aware of their symptoms of pain and depression but least aware of their decreased libido (Fig. 3).

Respondents experienced symptoms of CS on 16 days, on average, in a typical month and saw their physicians in the outpatient setting, on average, 6 times per year (Table 3). The 27 respondents who were employed at the time of the survey reported missing 2 days of work per month, or approximately 25 days per year, due to CS.

**Table 3:** Economic Impacts of CS in the Past 12 Months

	Frequency in Past 12 Months Mean ± SD (N = 55)
Outpatient visits	6.1 ± 2.9
Inpatient visits	
- Monitoring - Treatment or Surgery	0.4 ± 0.9 0.7 ± 1.3
Emergency room visits	0.5 ± 0.9
Symptom-free days	13.8 ± 9.7
Missed workdays^1^	2.1 ± 1.8 (N = 21)

^1^Missed workdays in past 30 days among patients who were currently employed

### Sub-group analysis

A sub-group analysis of respondents by surgical status revealed those who did not receive surgery (n = 11) scored significantly worse (p < 0.05) on almost all HRQoL parameters than respondents who did receive surgery (n = 44) including overall HRQoL and the CushingQoL scale, number of symptom-free days in the last month, missed workdays in the past 30 days, impact on work productivity in the last 30 days, VAS pain scores, severe fatigue measured by the validated BFI scale, and anxiety measured by the validated PROMIS Anxiety SF scale. Sleep scores were the only parameter tested that was not statistically significantly different between surgical and non-surgical subgroups.

## Discussion

This study was designed to evaluate the BOI of CS in order to enhance our knowledge of the patient experience and identify areas of unmet need. Despite previous research on the disease burden of CS, the holistic burden has not been well described in the published medical literature to date. [20] CS is characterized by a constellation of symptoms that overlap with symptoms experienced by other patient populations and requires a tailored research approach. Generic PRO instruments can address important symptoms, but may lack the specificity needed to fully profile the burden of the clinically complex symptom profile seen in conditions like CS. While the CushingQoL and Tuebingen CD-25 questionnaire have been developed as validated PRO measures designed specifically for CS and CD respectively, the lack of available normative values for these scales prevents comparison to other populations. [21] By combining a CS-symptom specific PRO with PROs for general use, our survey generated data that facilitates comparisons to other patients with CS, and possibly to other patient populations and the general population. To the authors’ knowledge, this is the first study of patients with endogenous CS to utilize multiple validated PROs in a single observational study to elucidate the significant, multi-factorial disease burden. Additionally, the longitudinal data (initial occurrence, time of diagnosis, and the time of the survey) reported in this study demonstrates a persistent BOI over time in patients with treated CS.

The demographics and characteristics of the respondent sample in this study (i.e., age, gender, disease duration, comorbidities, and previous treatments) reflected reported characteristics of patients with CS. [1, 3, 22–28] The sample in the study, due to inclusion criteria, also all had a history of pharmacotherapy treatment. While not typical of the greater CS patient population, this is an important patient cohort as recurrent CS is a common occurrence that endocrinologists face in clinical practice. In current literature, recurrence rates of CS can range up to 35% indicating the need for medical options including pharmacotherapies for this patient population. [29].

Patients with CS face a multi-faceted BOI that adversely impacts their HRQoL and is associated with healthcare utilization costs and lost productivity. This burden is increased by diagnostic challenges that often prevent a prompt diagnosis and by a perceived lack of physician knowledge or understanding of the condition, reported by more than 1 in 3 patients, which could result in exacerbated symptoms. The diagnosis of CS in this cohort was delayed by an average of 10 years from symptom onset to confirmed diagnosis which is longer than the 2–3 year delay reported in the current literature. [3] There was also a high symptomatic burden at the time of first occurrence of symptoms that extends into the current time, following treatment. Prolonged exposure to excess cortisol, such as that experienced by patients in the interval between onset of CS and diagnosis, may contribute to an increase in glucocorticoid withdrawal symptoms following CS treatment, which may further increase disease burden. [30] Respondents also reported that at the current time, sexual activity level was the daily activity most impacted by their CS, but out of all of their symptoms of CS, their physicians were least aware of the impact of decreased libido. This disconnect between physicians’ perceptions and patients’ reality may be a barrier to providing the best possible care.

All respondents in this study received at least one course of pharmacologic treatment for their CS, and most had surgery. Our results showed that most patient-reported symptoms declined in intensity and frequency from the first occurrence to the time of the survey, but not to the level of statistical significance. These findings are in accordance with previous research showing improvements in patient HRQoL following treatment, however, anxiety and pain were largely unchanged. [5, 11, 31] This finding is consistent with previous studies that have reported on the persistence of mental health and cognitive symptoms after successful biochemical resolution of CS. [5, 31, 32] However comparable data on pain is sparse in the published literature. Such persistent symptoms illustrate the durable nature of CS symptoms, including mood disorders, that are not adequately addressed by available treatments and are likely exacerbated by the diagnostic challenges and delays discussed earlier.

Validated PRO scales can be used in both the general population and those with chronic conditions allowing comparisons across populations. The validated PRO scales utilized in this study demonstrated moderate HRQoL interference, moderate to severe fatigue in most patients, and the presence of mild pain. The overall BOI, as manifested by multiple persistent symptoms, continues despite treatment to address the underlying hypercortisolism. [5, 11–13, 21, 31–33].

The treatment landscape for CS has evolved significantly in the last decade and there is recent emphasis on the use of PROs to guide clinical decision making and facilitate patient centered care. Attention has been placed on the benefits of PROs for informing safety and tolerability of treatments in clinical trials. [34] Use of PRO measures, particularly in head-to-head clinical trials in CS, can provide important information on the impact of various medical therapies, HRQoL, and may help guide pharmacologic decision making. [35] Given the symptomatic complexity of CS, the longitudinal use of a curated cadre of PRO measures may allow physicians to capture patient BOI more effectively and detect early recurrence. Electronic health platforms and patient portals could be used to facilitate the real-world implementation of this strategy.

Finally, the economic aspect of the burden of CS was explored starting with HCRU as a direct economic cost. Our results showed respondents averaged over 6 outpatient visits annually for their condition, more than twice as many as the average American. [36] This number is also comparable to previous studies, which have reported higher rates of HCRU among patients with CS than matched controls including 3 to 10 CS-related outpatient visits per year, and represents a substantial ongoing burden for the healthcare system in direct costs. [22, 23, 37] Additionally, the time to reach and receive this care, as well as the logistical burden of coordinating frequent care, may also adversely impact patients with CS.

This survey also assessed work productivity to gather information on the indirect economic costs of CS. Despite a mean age of 43 years, about half of the respondents in this study were not employed at the time of the survey, which suggests CS may present a barrier to employment. Respondents who were employed at the time of the survey (n = 27) reported missing an average of over 2 workdays in the preceding month or about 25 days per year due to CS. This markedly exceeds the US population average of 4 missed workdays per year due to illness or injury (excluding maternity leave) and demonstrates that even among the minority of respondents able and willing to work full time, CS presented a substantial burden hampering their work productivity and limiting their economic potential. [38].

Our results reinforce and expand on earlier work on the ongoing burden of CS from the perspective of the patient and illustrate the need for interventions to address persistent symptoms, such as weight gain, a symptom that was prevalent over the course of the disease journey. This work can pave the way for more rigorous research on CS related symptoms in treated patients. Future patient-centered research in CS should focus on ensuring the appropriate level of context is captured, including CS-specific symptom assessments and associated population normative values, to maximize the usefulness of instruments like the CushingQoL. In addition, assessing the symptom specific burden of disease and HRQoL using generic instruments may help to inform the overall disease burden as well as bridge the gap between patient and physician perceptions.

Finally, future studies should assess concomitant medications used to address symptoms of CS, which may reveal symptomatic burdens potentially masked in the current data. Our study captured information on concomitant use of OTC analgesics, showing that nearly 9 in 10 respondents used them regularly, and it has been previously reported that antidepressant use in this population is higher than the general population even after diagnosis and remission of other symptoms. [39] Better data on prescription and OTC medications used to treat the symptoms of CS as well as other contextual details could also help refine our current understanding of the ongoing BOI of CS.

There are several limitations to this study, which should be considered when interpreting the results. CS is a rare disease, so it was a challenge to identify and enroll eligible patients. Our sample also had a higher percentage of patients with ACTH-independent CS than typically observed in the population. The sample size used in this study, 55, limited the power of some statistical analyses. This study also used convenience sampling, which involves recruiting patients willing and able to participate in an online survey. Responses may have been subject to recall bias if respondents were not able to accurately remember previous events or experiences, omitted details, or whose memories were influenced by subsequent events and experiences. For one, patients may have misclassified the etiology of their CS. In addition, responses to the web-enabled questionnaire may have been subject to social desirability bias, which would result in underreporting of socially undesirable attitudes and overreporting of socially desirable attributes. This study included a group of CS patients who were more likely to have either failed standard first line therapy or were not candidates for it, which was why they were being treated medically. The BOI in this cohort may therefore be greater than the BOI in patients who achieve biological remission after first line therapy and never require medical treatment.

Despite these limitations, the respondents in this study were comparable in age and gender distribution to the overall population with CS, which supports the relevance of these results. It is important to note the survey was fielded during the COVID-19 pandemic, so activities such as travel and possibly employment may have been affected independent of disease status. Finally, the survey did not capture symptom-specific pharmacologic treatments except for OTC analgesics. A lack of data regarding use of other treatments such as sleep aids could be masking the true symptom burden in this patient sample.

Overall, these data identify a continued high BOI in treated endogenous CS patients and highlight an unmet need for interventions that address their symptomatic burden. Our results illustrate the need for interventions to address persistent symptoms and underscore the importance of the patient’s perspective in clinical decision making and the development of therapeutics for Cushing’s syndrome.

## Data Availability

The authors confirm that all pertinent data generated or analyzed during this study are included in this manuscript or supplementary materials.
